# Early postoperative hyperglycaemia is not a risk factor for infectious complications and prolonged in-hospital stay in patients undergoing oesophagectomy: a retrospective analysis of a prospective trial

**DOI:** 10.1186/cc2970

**Published:** 2004-10-18

**Authors:** Titia M Vriesendorp, J Hans DeVries, Jan BF Hulscher, Frits Holleman, Jan J van Lanschot, Joost BL Hoekstra

**Affiliations:** 1 Research Physician, Department of Internal Medicine, Academic Medical Centre, Amsterdam, The Netherlands; 2 Internist-Endocrinologist, Department of Internal Medicine, Academic Medical Centre, Amsterdam, The Netherlands; 3 Surgical Resident, Department of Surgery, Academic Medical Centre, Amsterdam, The Netherlands; 4 Professor of Surgery, Department of Surgery, Academic Medical Centre, Amsterdam, The Netherlands; 5 Professor of Internal Medicine, Department of Internal Medicine, Academic Medical Centre, Amsterdam, The Netherlands

**Keywords:** hyperglycaemia, infection, length of stay, oesophagectomy, risk factor

## Abstract

**Introduction:**

Treating hyperglycaemia in hospitalized patients has proven to be beneficial, particularly in those with obstructive vascular disease. In a cohort of patients undergoing resection for oesophageal carcinoma (a group of patients with severe surgical stress but a low prevalence of vascular disease), we investigated whether early postoperative hyperglycaemia is associated with increased incidence of infectious complications and prolonged in-hospital stay.

**Methods:**

Postoperative glucose values up to 48 hours after surgery were retrieved for 151 patients with American Society of Anesthesiologists class I or II who had been previously included in a randomized trial conducted in a tertiary referral hospital. Multivariate regression analysis was used to define the independent contribution of possible risk factors selected by univariate analysis.

**Results:**

In univariate regression analysis, postoperative glucose levels were associated with increased length of in-hospital stay (*P *< 0.001) but not with infectious complications (*P *= 0.21). However, postoperative glucose concentration was not found to be an independent risk factor for prolonged in-hospital stay in multivariate analysis (*P *= 0.20).

**Conclusion:**

Our data indicate that postoperative hyperglycaemia is more likely to be a risk marker than a risk factor in patients undergoing highly invasive surgery for oesophageal cancer. We hypothesize that patients with a low prevalence of vascular disease may benefit less from intensive insulin therapy.

## Introduction

Until recently hyperglycaemia after surgery was considered to be a benign phenomenon. However, in a landmark study, van den Berghe and coworkers [1] showed that treating transient postoperative hyperglycaemia with intensive insulin therapy in a surgical intensive care unit (ICU) dramatically reduces mortality and morbidity. Strict glucose control (target range between 4.4 mmol/l and 6.1 mmol/l) was responsible for a reduction in both ICU and in-hospital mortality, which was primarily attributed to the prevention of septic complications [1].

The population studied by van den Berghe and coworkers was diverse but consisted primarily of patients who underwent cardiac surgery (63%). Others have found beneficial effects of intensive insulin therapy in patients with obstructive vascular disease such as acute myocardial infarction and acute stroke, and in those who have undergone cardiovascular bypass surgery [2-7]. Strict glucose control is relatively time consuming for ICU personnel because of frequent glucose monitoring, and it may be hazardous because of the risk for hypoglycaemia. It is therefore important to determine which patient groups in the ICU are likely to benefit most or least from aggressively correcting hyperglycaemia.

We investigated whether postoperative hyperglycaemia is a risk factor for postoperative infections and prolonged in-hospital stay in a cohort of patients undergoing resection for adenocarcinoma of the oesophagus (i.e. patients with a low prevalence of risk factors for insulin resistance and cardiovascular disease but who are subject to great postoperative stress).

## Methods

### Patients

A total of 220 consecutive patients with adenocarcinoma of the oesophagus from two university hospitals in Amsterdam and Rotterdam were included in a previously reported randomized clinical trial investigating differences in short-term and long-term morbidity and mortality between two surgical approaches for resection of oesophageal adenocarcinoma [8]. Classification into American Society of Anesthesiologists (ASA) class 1 or 2 was a requirement for eligibility in that study.

Only patients included in Amsterdam were included in the present analysis (*n *= 160), because glucose values were taken only in a small proportion of the Rotterdam patients. In nine cases oesophageal resection was cancelled peroperatively because of distant dissemination of tumour, leaving 151 patients for this analysis.

### Data collection

Glucose values were automatically determined with each arterial blood sample test (Ciba Corning 865; Chiron Diagnostics, Medford, MA, USA), and were collected retrospectively from laboratory reports. Forced expiratory volume in 1 s (FEV_1_) expressed as percentage of the predicted value corrected for age and sex, and patient height (to calculate body mass index [BMI]) were collected retrospectively from preoperative lung function reports. Insulin use in the first 48 hours after surgery was determined retrospectively from ICU charts. In the prospective cohort patients were visited at least twice a week by one of the investigators to score postoperative complications. Postoperative infections were defined as signs of infection and positive culture [9]. History of cardiovascular disease, hypertension, weight loss, ASA class, postoperative occurrence of left ventricular failure and length of hospital stay were determined prospectively [8].

Patients were allowed to eat as they wished until 24 hours before surgery. Patients with more than 10% weight loss in the year preceding surgery received preoperative enteral tube feeding. Postoperatively, all patients received continuous tube feeding through a needle jejunostomy, starting 12–24 hours postoperatively, with 25 ml/hour tube feeding containing immunomodulatory nutrients (Impact^®^; Novartis, Basel, Switserland). As a general rule, patients received 30 ml glucose 5% intravenously during the first 48 hours after surgery and patients were treated with insulin when glucose values exceeded 12 mmol/l.

### Statistical analysis

For each patient the mean postoperative glucose concentration was calculated using all available glucose measurements obtained until 48 hours postoperatively. For further analysis, mean postoperative glucose concentrations were divided into quartiles because of nonparametric distribution.

Univariate regression analysis was used to select parameters associated with infectious complications and length of hospital stay. Parameters with *P *< 0.1 in univariate regression analysis were examined in multivariate analysis to define the independent contribution of each possible risk factor [10]. Postoperative glucose concentrations were automatically selected for multivariate analysis because it was the main aim of the study to determine their relationship with outcome. Logistic regression analysis was used for infectious complications, and linear regression analysis was used for length of stay. Because of nonparametric distribution, length of stay data were logarithmically transformed before regression analysis.

### Parameters included in the analysis

Age, amount of preoperative weight loss, BMI and FEV_1 _were entered into regression analyses as continuous variables. Postoperative glucose levels, insulin use within 48 hours after surgery, type of surgical procedure, sex, ASA class, history of hypertension, coronary artery disease, cardiac valve disease or arrhythmia, clinical staging of the tumour and presence of diabetes mellitus were entered as categorical variables.

## Results

Preoperative characteristics are summarized in Table 1. At least one postoperative glucose value could be retraced in 150 out of 151 cases (99%; median 7 glucose values per patient; range 1–21). A glucose level greater than 6.1 mmol/l was found in 97% of patients. During the first 48 hours after surgery, insulin was administrated to four patients with known diabetes mellitus and to five patients without diabetes mellitus, but insulin administration could not be retraced in one patient with known diabetes mellitus. At least one infectious complication occurred in 55 patients (36%) and more than one infection occurred in 15 patients (9.9%). Pneumonia occurred in 44 patients, wound infection in 15, urinary tract infection in six and sepsis in seven. Patients were admitted to the ICU for a median duration of 3 days (range <24 hours to 71 days). The median length of stay was 16 days (range 9–154 days). The incidences of postoperative left ventricular failure (*n *= 13; 8.6%) and in-hospital death (*n *= 5; 3.3%) were too low to allow for regression analysis.

### Postoperative glucose levels and postoperative infections

**Figure 1:**
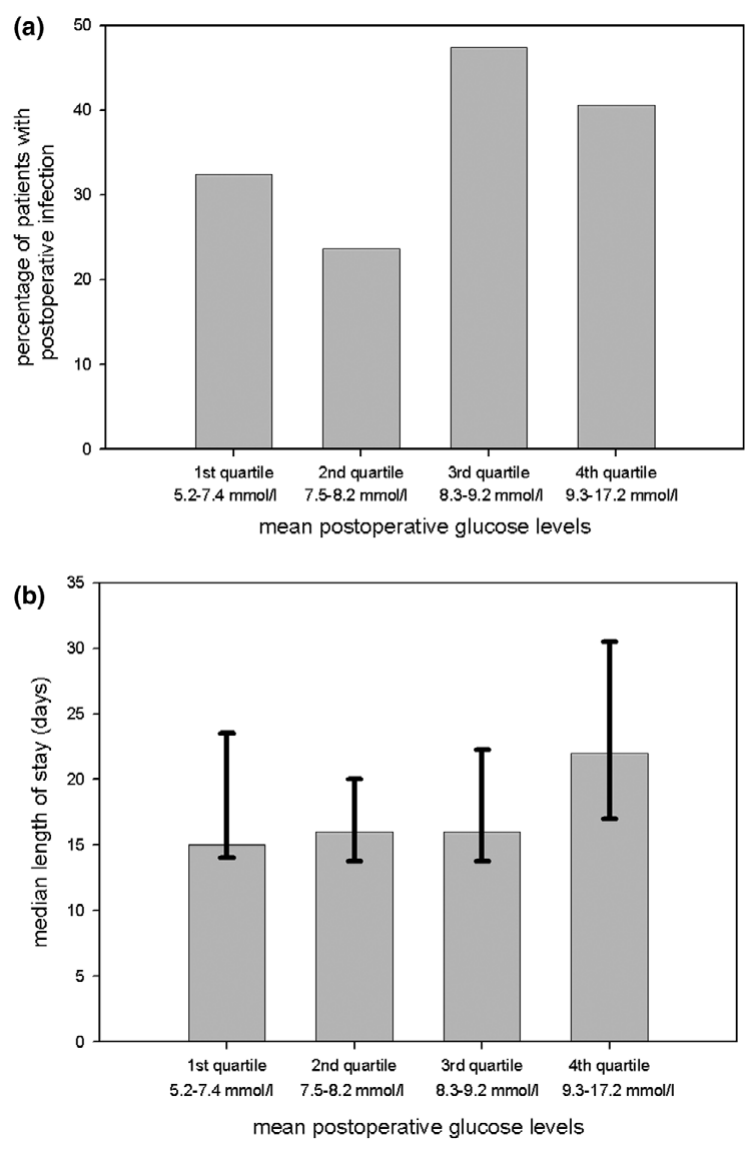
Percentage of **(a) **infections and **(b) **median length of hospital stay per glucose quartile: first quartile 5.2–7.4 mmol/l, second quartile 7.5–8.2 mmol/l, third quartile 8.3–9.2 mmol/l, and fourth quartile 9.3–17.2 mmol/l. The error bars in panel b represent the interquartile range.

According to univariate regression analysis, no association was found between postoperative glucose levels and infectious complications (*P *= 0.21; Fig. 1a) or between insulin administration and infectious complications (*P *= 0.37; odds ratio [OR] 0.5, 95% confidence interval [CI] 0.1–2.4). Parameters associated with postoperative infections in univariate regression analysis were history of cardiac valve disease or arrhythmia (*P *= 0.026; OR 11.5, 95% CI 1.35–98.2), FEV_1 _per 10% increase (*P *= 0.021; OR 0.78, 95% CI 0.63–0.96; OR per 10% of expected FEV_1_), age per 10 years (*P *= 0.069; OR 1.39, 95% CI 0.98–1.97) and duration of surgery per hour (*P *= 0.059; OR 1.23, 95% CI 0.99–1.52). In the subgroup of patients with an ICU stay in excess of 5 days, there was no association between postoperative hyperglycaemia and infection (*P *= 0.9 for trend; *P *= 0.8 by ?^2 ^analysis). Also in multivariate analysis, postoperative hyperglycaemia was not found to be a predictor of postoperative infection (*P *= 0.28; OR 1.21, 95% CI 0.86–1.72; Table 2). Also, patients with at least one glucose value in excess of 10 mmol/l were not at greater risk for infections (data not shown).

### Postoperative glucose levels and length of stay

In univariate analysis, a positive association was found between postoperative hyperglycaemia and length of hospital stay (P < 0.001; ß = 0.053; standard error [SE] of ß = 0.014), but not with insulin administration (*P *= 0.5; ß = -0.56; SE of ß = 0.7). Other parameters associated with length of in-hospital stay were duration of surgery (*P *< 0.001; ß = 0.050; SE of ß = 0.010), transthoracic procedure (*P *< 0.001; ß = 0.119, SE of ß = 0.032), BMI (*P *= 0.036; ß = 0.013; SE of ß = 0.006) and history of cardiac valve disease or arrhythmia (*P *= 0.103; ß = 0.130; SE of ß = 0.079). After correction for these variables in multivariate analysis, mean postoperative glucose concentration was found not to be an independent risk factor for prolonged hospital stay (*P *= 0.20; Table 3). Adding duration of ICU stay greater than 5 days as an interaction term was not statistically significant (*P *= 0.12).

## Discussion

In a cohort of patients undergoing highly invasive surgery for oesophageal cancer, we found that postoperative hyperglycaemia was present in almost all patients but that it was not associated with increased incidence of postoperative infections and length of hospital stay.

Van den Berghe and coworkers [1] found that lowering postoperative hyperglycaemia with intensive insulin therapy significantly decreased morbidity and mortality in postoperative patients. *Post hoc *analysis revealed that both administration of insulin and, possibly to a greater degree, lower glucose levels contributed to better outcome [11]. However, it is unclear how the effect of intensive insulin therapy in surgical intensive care patients can be explained and which patient groups benefit most from intensive insulin therapy. We propose the following explanation for the seemingly contradictory findings of our study.

The population evaluated in the study by van den Berghe and coworkers [1] consisted mainly of patients undergoing cardiovascular surgery. Transient or 'stress induced' hyperglycaemia was previously reported to be associated with a poor prognosis, primarily in patients with obstructive vascular disease such as those with acute myocardial infarction and acute stroke, and in those who have undergone cardiovascular bypass surgery and peripheral vascular surgery [12-16]. Few patients in our cohort suffered from (cardio)vascular disease because ASA class 1 or 2 was a prerequisite for inclusion in the study, and only 11% had a history of coronary artery disease. It could thus be hypothesized that, in a population with little vascular disease, high postoperative glucose levels are not associated with poor outcome.

In response to surgery, both plasma glucose levels and free fatty acid (FFA) levels rise [17]. Pathophysiological mechanisms that may explain the relationship between stress induced hypermetabolism and poor outcome in patients with cardiovascular disease include the following: toxic effects of elevated FFA levels on the ischaemic myocardium [18]; elevated FFA levels and hyperglycaemia causing QT prolongation [19]; hyperglycaemia attenuating ischaemic preconditioning [20]; and hyperglycaemia causing reduced collateral coronary perfusion [21]. Haemodynamic effects of glucose and insulin may also play an important role in the pathophysiology of stress induced hypermetabolism. Hyperglycaemia has vasoconstrictive effects [22], which may aggravate tissue ischaemia, particularly in patients with obstructive vascular disease. Insulin has been reported to have vasodilatory effects, and part of the beneficial effect of intensive insulin therapy may be explained by increasing tissue perfusion [23].

Our data do not exclude the possibility that intensive insulin therapy or glucose–insulin–potassium infusions may still be beneficial in this particular subgroup of patients. The benefits of intensive insulin therapy may not solely be attributed to lowering hyperglycaemia, but may be mediated by the effect of insulin on protein and lipid metabolism, independent of its effects on glucose metabolism. In patients with sepsis and cancer, lower levels of insulin are needed to restore lipid levels than glucose levels [24]. Similarly, depleted protein storage and severe surgical stress after oesophageal resection may impair the immune response postoperatively and thus increase the risk for postoperative infection [25], which may be ameliorated by insulin. However, the administration of insulin was not associated with lower infection risk in our cohort.

A shortcoming of the present study is that the number of glucose measurements taken in each patient was not standardized, because of the study's retrospective design. For some patients more glucose measurements were available than for others, and this may have influenced our results. However, glucose measurements were taken randomly with each arterial blood gas analysis, and because mean postoperative glucose levels were used, the relative weight of incidental extreme values was diminished. A strength of our cohort is its homogeneity. It represents a unique group of patients with high postoperative stress and a low frequency of risk factors for obstructive vascular disease.

## Conclusion

Despite the limitations associated with the retrospective analysis of a prospective study, our data indicate that early postoperative hyperglycaemia is more likely to be a risk marker than a risk factor in a patient group encountering severe surgical stress but with a low prevalence of cardiovascular disease. We therefore suggest that the value of intensive insulin therapy, which is time consuming and potentially hazardous, needs further investigation in this particular patient group.

## Key messages

• 	Postoperative hyperglycaemia after oesophagectomy was not found to be associated with postoperative infection risk.

• 	Postoperative hyperglycaemia after oesophagectomy was found to be associated with longer duration of postoperative stay. However, when corrected for possible confounders, postoperative hyperglycaemia was not found to be an independent risk factor for longer duration of stay.

• 	Strict glycaemic control may not be beneficial for patients after oesophagectomy.

## Competing interests

The author(s) declare that they have no competing interests.

## Author contributions

TMV participated in the design of the study, data collection, data analysis and writing of the manuscript. JHDV participated in data analysis and writing of the manuscript. JBH participated in the design of the study, data collection, data analysis and writing of the manuscript. FH participated in the design of the study and writing of the manuscript. JJvL participated in the design of the study, data collection and writing of the manuscript. JBLH participated in the design of the study, writing of the manuscript and coordinated the study.

## Abbreviations

ASA = American Society of Anesthesiologists; BMI = body mass index; CI = confidence interval; FEV_1 _= forced expiratory volume in 1 s; FFA = free fatty acid; ICU = intensive care unit; OR = odds ratio; SE = standard error.

## Figures and Tables

**Table 1:** Other possible risk factors for infection and length of stay

Preoperative parameter	Value	Missing values (%)
Age in years (mean ± SD)	62.4 ± 10.0	0
Male sex (*n *[%])	127 (84.1)	0
ASA		0
Class I (*n *[%])	47 (31.1)	
Class II (*n *[%])	104 (68.9)	
History of cardiac valve disease or arrhythmia (*n *[%])	7 (4.6)	0.7
History of hypertension (*n *[%])	21 (13.9)	0.7
History of coronary artery disease (*n *[%])	16 (10.6)	0.7
BMI (mean ± SD)	25.4 ± 3.3	25.8
Percentage of expected FEV_1 _(mean ± SD)	101 ± 18	18.5
Diabetes mellitus (*n *[%])	9 (6.0)	0.7
Preoperative weight loss (kg; mean ± SD)	5.3 ± 6.6	7.9
Clinical staging of tumor		1.3
Stage I (*n *[%])	18 (11.9)	
Stage II (*n *[%])	68 (45.0)	
Stage III (*n *[%])	58 (38.4)	
Stage IV (*n *[%])	5 (3.3)	
Allocated to transthoracic procedure (*n *[%])	78 (51.7%)	0
Duration of surgery (hours; mean ± SD)	5.3 (1.6)	0.7
Insulin use within 48 hours after surgery (*n *[%])	9 (6.0%)	9.2

A total of 151 patients were included. ASA, American Society of Anesthesiologists; BMI, body mass index; FEV_1_, forced expiratory volume in 1 s; SD, standard deviation.

**Table 2:** Multivariate analysis of infectious complications

Prognostic variable	OR (95% CI)	*P*
FEV_1 _(per 10% of expected FEV_1_)	0.79 (0.63–0.99)	0.045
History of cardiac valve disease or arrhythmia	7.30 (0.78–68.3)	0.081
Duration of surgery	1.27 (0.98–1.64)	0.069
Age per 10 years	1.36 (0.90–2.07)	0.142
Mean postoperative glucose	1.21 (0.86–1.72)	0.279

CI, confidence interval; FEV1, forced expiratory volume in 1 s; OR, odds ratio.

**Table 3:** Multivariate analysis of length of stay

Prognostic variable	ß	SE of ß	*P*
Duration of surgery	0.062	0.021	0.004
BMI	0.010	0.006	0.072
Mean postoperative glucose	0.024	0.018	0.195
History of cardiac valve disease or arrhythmia	0.058	0.091	0.527
Transhiatal procedure	0.034	0.065	0.599

BMI, body mass index; SE, standard error.

## References

[B1] van den Berghe G, Wouters P, Weekers F, Verwaest C, Bruyninckx F, Schetz M, Vlasselaers D, Ferdinande P, Lauwers P, Bouillon R (2001). Intensive insulin therapy in critically ill patients. N Engl J Med.

[B2] Malmberg K (1997). Prospective randomised study of intensive insulin treatment on long term survival after acute myocardial infarction in patients with diabetes mellitus. DIGAMI (Diabetes Mellitus, Insulin Glucose Infusion in Acute Myocardial Infarction) Study Group. BMJ.

[B3] Lazar HL, Chipkin SR, Fitzgerald CA, Bao Y, Cabral H, Apstein CS (2004). Tight glycemic control in diabetic coronary artery bypass graft patients improves perioperative outcomes and decreases recurrent ischemic events. Circulation.

[B4] Diaz R, Paolasso EA, Piegas LS, Tajer CD, Moreno MG, Corvalan R, Isea JE, Romero G (1998). Metabolic modulation of acute myocardial infarction. The ECLA (Estudios Cardiologicos Latinoamerica) Collaborative Group. Circulation.

[B5] van der Horst IC, Zijlstra F, van't Hof AW, Doggen CJ, de Boer MJ, Suryapranata H, Hoorntje JC, Dambrink JH, Gans RO, Bilo HJ, Zwolle Infarct Study Group (2003). Glucose-insulin-potassium infusion inpatients treated with primary angioplasty for acute myocardial infarction: The glucose-insulin-potassium study: a randomized trial. J Am Coll Cardiol.

[B6] Scott JF, Robinson GM, French JM, O'Connell JE, Alberti KG, Gray CS (1999). Glucose potassium insulin infusions in the treatment of acute stroke patients with mild to moderate hyperglycemia: the Glucose Insulin in Stroke Trial (GIST). Stroke.

[B7] Furnary AP, Zerr KJ, Grunkemeier GL, Starr A (1999). Continuous intravenous insulin infusion reduces the incidence of deep sternal wound infection in diabetic patients after cardiac surgical procedures. Ann Thorac Surg.

[B8] Hulscher JB, van Sandick JW, de Boer AG, Wijnhoven BP, Tijssen JG, Fockens P, Stalmeier PF, ten Kate FJ, van Dekken H, Obertop H (2002). Extended transthoracic resection compared with limited transhiatal resection for adenocarcinoma of the esophagus. N Engl J Med.

[B9] van Sandick JW, Gisbertz SS, ten Berge IJ, Boermeester MA, van der Pouw Kraan TC, Out TA, Obertop H, van Lanschot JJ (2003). Immune responses and prediction of major infection in patients undergoing transhiatal or transthoracic esophagectomy for cancer. Ann Surg.

[B10] Katz MH (2003). Multivariable analysis: a primer for readers of medical research. Ann Intern Med.

[B11] Van den Berghe G, Wouters PJ, Bouillon R, Weekers F, Verwaest C, Schetz M, Vlasselaers D, Ferdinande P, Lauwers P (2003). Outcome benefit of intensive insulin therapy in the critically ill: Insulin dose versus glycemic control. Crit Care Med.

[B12] Latham R, Lancaster AD, Covington JF, Pirolo JS, Thomas CS (2001). The association of diabetes and glucose control with surgical-site infections among cardiothoracic surgery patients. Infect Control Hosp Epidemiol.

[B13] McAlister FA, Man J, Bistritz L, Amad H, Tandon P (2003). Diabetes and coronary artery bypass surgery: an examination of perioperative glycemic control and outcomes. Diabetes Care.

[B14] Norhammar AM, Ryden L, Malmberg K (1999). Admission plasma glucose. Independent risk factor for long-term prognosis after myocardial infarction even in nondiabetic patients. Diabetes Care.

[B15] Capes SE, Hunt D, Malmberg K, Gerstein HC (2000). Stress hyperglycaemia and increased risk of death after myocardial infarction in patients with and without diabetes: a systematic overview. Lancet.

[B16] Vriesendorp TM, Morelis QJ, DeVries JH, Legemate DA, Hoekstra JB (2004). Early postoperative glucose levels are an independent risk factor for infection after peripheral vascular surgery. A retrospective study. Eur J Vasc Endovasc Surg.

[B17] Frayn KN (1986). Hormonal control of metabolism in trauma and sepsis. Clin Endocrinol (oxf).

[B18] Oliver MF, Opie LH (1994). Effects of glucose and fatty acids on myocardial ischaemia and arrhythmias. Lancet.

[B19] Marfella R, Nappo F, De Angelis L, Siniscalchi M, Rossi F, Giugliano D (2000). The effect of acute hyperglycaemia on QTc duration in healthy man. Diabetologia.

[B20] Kersten JR, Schmeling TJ, Orth KG, Pagel PS, Warltier DC (1998). Acute hyperglycemia abolishes ischemic preconditioning in vivo. Am J Physiol.

[B21] Kersten JR, Toller WG, Tessmer JP, Pagel PS, Warltier DC (2001). Hyperglycemia reduces coronary collateral blood flow through a nitric oxide-mediated mechanism. Am J Physiol Heart Circ Physiol.

[B22] Giugliano D, Marfella R, Coppola L, Verrazzo G, Acampora R, Giunta R, Nappo F, Lucarelli C, D'Onofrio F (1997). Vascular effects of acute hyperglycemia in humans are reversed by L-arginine. Evidence for reduced availability of nitric oxide during hyperglycemia. Circulation.

[B23] Yki-Jarvinen H, Utriainen T (1998). Insulin-induced vasodilatation: physiology or pharmacology?. Diabetologia.

[B24] Sauerwein HP, Pesola GR, Groeger JS, Jeevanandam M, Brennan MF (1988). Relationship between glucose oxidation and FFA concentration in septic cancer-bearing patients. Metabolism.

[B25] Tashiro T, Yamamori H, Takagi K, Hayashi N, Furukawa K, Nitta H, Toyoda Y, Sano W, Itabashi T, Nishiya K (1999). Changes in immune function following surgery for esophageal carcinoma. Nutrition.

